# Agile Delphi methodology: A case study on how technology impacts burnout syndrome in the post-pandemic era

**DOI:** 10.3389/fpubh.2022.1085987

**Published:** 2023-01-20

**Authors:** Fuensanta Medina-Dominguez, Maria-Isabel Sanchez-Segura, Antonio de Amescua-Seco, Germán-Lenin Dugarte-Peña, Santiago Villalba Arranz

**Affiliations:** ^1^Computer Science and Engineering Department, Universidad Carlos III de Madrid, Leganés, Madrid, Spain; ^2^Universidad Francisco de Vitoria, Madrid, Spain; ^3^Unidad Técnica de Diseño, Innovación y Desarrollo, Instituto Regional de Seguridad y Salud en el Trabajo de la Comunidad de Madrid, Madrid, Spain

**Keywords:** burnout syndrome, Occupational Health and Safety, Delphi method, human and organizational factors, Safety

## Abstract

**Introduction:**

In the post-pandemic era, many habits in different areas of our lives have changed. The exponential growth in the use of technology to perform work activities is one of them. At the same time, there has been a marked increase in burnout syndrome. Is this a coincidence? Could they be two interconnected developments? What if they were? Can we use technology to mitigate this syndrome? This article presents the agile Delphi methodology (MAD), an evolved version of the Delphi method, adapted to the needs of modern-day society.

**Methods:**

To drive Occupational Health and Safety (OHS) experts to reach a consensus on what technological and non-technological factors could be causing the burnout syndrome experienced by workers in the post-pandemic era, MAD has been used in a specific case study. This study formally presents MAD and describes the stages enacted to run Delphi experiments agilely.

**Results:**

MAD is more efficient than the traditional Delphi methodology, reducing the time taken to reach a consensus and increasing the quality of the resulting products.

**Discussion:**

OHS experts identified factors that affect and cause an increase in burnout syndrome as well as mechanisms to mitigate their effects. The next step is to evaluate whether, as the experts predict, burnout syndrome decreases with the mechanisms identified in this case study.

## 1. Introduction

It is difficult to ignore the extreme social and economic shake-up that we have been experiencing since early 2020. The global health crisis caused by COVID-19, which has enveloped the entire planet, has changed our work habits, among many other things. According to Moss (1), in April 2020, 81% of workplaces were closed, leaving 2.6 billion people (knowledge workers) locked up and working from home. However, not only did they lock themselves up to work but they also spent their leisure time online, that is, first-world citizens were suddenly living their whole life through information and communication technologies. An example was the growth in popularity of the Zoom platform, which increased from 10 million to 200 million daily users over a few months.

In 2018, the Spanish National Institute for Occupational Health and Safety developed an interesting study (2) to analyze the psychosocial impact of the use of information and communication technologies (ICT) in the workplace. This study concluded that they had both positive and negative effects but, of course, did not account for the situation experienced over the last few years as a result of the COVID-19 pandemic. Although the situation is no longer as extreme as it was in 2020, COVID-19 certainly triggered and left important changes in its wake that have made us consider our hyperconnected society and its potential connection to the exponential increase in one of the most important psychosocial risks that affect workers, which is the well-known burnout syndrome.

This is not a new syndrome. Much has been written about burnout syndrome since 1974, but it was not until 2019 that it was finally included by the World Health Organization (WHO) in its 10th revision of the International Classification of Diseases (ICD-10) and described as *a syndrome conceptualized as a result of chronic work-related stress that has not been successfully managed*. It is now classified in the 11th revision of the International Classification of Diseases (ICD-11) as an occupational phenomenon (3).

Although different methods for measuring burnout syndrome have been used since 1981 (4), the landscape has changed a great deal since then. We want to put it on the table and reanalyze this psychosocial risk considering the new landscape left behind by the precipitous change suffered as a consequence of the above health situation. We aimed to explore a potential relationship between burnout syndrome and the use of digital technologies, given the exponential growth that both are experiencing. This interest raises the following questions: Could the prominent role of technology in the lives of working people by increasing the number of people suffering from this syndrome? If this is the case and we cannot avoid the use of technology, what could we do to make technology less of a problem? What could we do to convert technology itself into an instrument to mitigate burnout syndrome?

Based on the reliability of its results, one of the best-known and most used techniques to determine whether or not there is consensus among a group of experts on certain criteria is the Delphi method (5). This method requires some adaptations to the agility of 21st-century demands for more efficient and effective adoption. Therefore, we present a series of adjustments made to this method in the “Research method” section. In the “Case study” section, we report the results of its application with the specific objective of determining whether or not there is consensus regarding the use of technology affecting workers in such a way as to cause burnout syndrome.

## 2. Literature review

This section first describes burnout syndrome and related works that pinpoint the factors and causes that may potentially exacerbate this syndrome, followed by the traditional Delphi methodology used as the baseline for this case study.

### 2.1. Burnout syndrome

The World Health Organization (WHO) announced burnout syndrome as the disease of the century. It is not a new syndrome. In 1974, psychiatrist Herbert J. Freudenberger started to investigate burnout. His studies focused mainly on the medical sector. He observed that his colleagues tended to lose empathy with their patients and suffer from exhaustion (6). The syndrome was, in its early days, related to the health and emergency sectors, such as police and firefighters. However, the syndrome is no longer confined to specific sectors (7, 8). There were early warning signs of burnout before the COVID-19 pandemic. However, during and after the pandemic, the incidence of burnout has increased significantly, and, consequently, the syndrome has come to be known as a “second” or “silent” pandemic (8).

In 2019, the WHO classified it as an occupational risk, and it was included in the International Statistical Classification of Diseases and Related Health Problems (ICD-11), which came into force on 1 January 2022 (8). The WHO defined burnout as *a syndrome conceptualized as resulting from chronic workplace stress that has not been successfully managed* (3). However, according to the foremost expert on burnout, Christina Maslach, the new WHO classification in the ICD-11 is concerning because *categorizing burnout as a disease was an attempt by the WHO to provide definitions for what is wrong with people instead of what is wrong with companies* (9). This should lead us to consider the organization that employs the worker, and not the worker, as responsible for burnout.

Additionally, when we analyzed the impact from not only the human (health) but also the economic point of view, we came across interesting statistics. In the United States, burnout costs $500 billion, and up to 550 million days are lost due to work stress. Every year in Europe, mental health costs between 3 and 5% of the GDP of the region. Burnout has a very big impact on the world economy, which is set to increase in proportion to the burnout trend.

To better understand the impact of burnout syndrome, several studies analyzed the factors that are affecting and causing an increase in employee burnout syndrome. Some of the identified factors are high-stress levels (10), workplace conflicts (11), social support among colleagues (12), job satisfaction (13), work–family reconciliation (14), the sense of control and autonomy (15), personal skills, training in communication skills (16), earnings from employment (17), unfair treatment at work, unmanageable workload, role ambiguity, deficiencies in communication and support from managers, unreasonable time pressure, and so on. However, none of these studies reflect technology as a cause of burnout. In contrast, the advantages of technology use at companies (productivity, efficiency, job satisfaction, etc.) are widely acknowledged (18). However, it was recently found that technology may also have a negative connotation and be synonymous with techno-stress, leading to burned-out employees whose performance drops (19–21).

The identification of the factors that increase burnout is just as important as analyzing the mechanisms that mitigate this syndrome. Therefore, we present a study carried out with experts in occupational risk prevention. We analyzed and explored the organizational factors that affect employees, increasing their burnout, as well as the mechanisms that they consider companies should implement to mitigate this syndrome.

Although there are other bodies of research identifying factors and methods, the difference and originality of this investigation in comparison to other studies with the same or similar objectives are that we used a technique called the Delphi method, where the participants, who are recognized experts with a lot of knowledge and experience on the subject, attempted to reach a consensus based on anonymous reflection and sharing of opinions.

### 2.2. Delphi method

The Delphi method was created in the United States in the 1950s. It has its origin in the philosophical field, where group knowledge is valued over individual knowledge, that is, *considering that the relevant information accumulated by a group of experts is always equal to or greater than that of the individual in particular* (5, 22). The Delphi method is based on the recognition of the superiority of group judgment over individual judgment. Therefore, the objective of the method is to obtain the most reliable consensus opinion from a group of experts (5).

The special characteristics of this method are as follows:

- Highly efficient expert selection process: A mechanism has been created to easily identify potential experts. Everyday jobs are performed by workers in physically distributed spaces. Therefore, it is very important to consider the opinions of a broad spectrum of experts from different countries and organizational cultures to assure the maximum possible diversity.
- Iterative process: The process is divided into several rounds. Participants express their opinions in each round. In between rounds, they have the opportunity to reflect on both their own opinions and those issued by the other experts.
- Regular feedback: The opinion of the experts on the problem being analyzed is communicated before each round.
- Anonymity: The experts do not know the source of each response. Anonymity has the advantage of preventing dominant members of the group from influencing or inhibiting other participants. Additionally, none of the experts communicate directly.
- Availability of group statistical results, if required. Statistics have been associated with response types to maximize efficiency and ensure that the results are analyzable.

**Figure 1:**
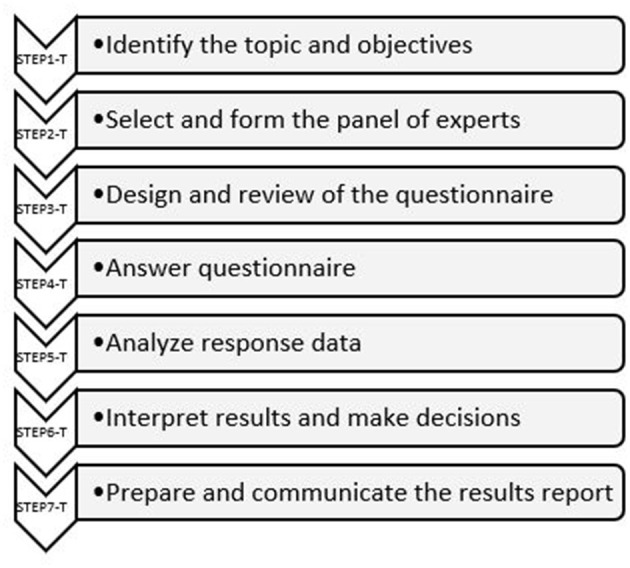
Traditional Delphi method.

Although there are different approaches to organizing Delphi activities (23–25), they all follow the same philosophy, consisting of the steps shown in Figure 1.

It is a methodology that is currently used in many scientific fields, like health sciences (26, 27), tourism (28), and emerging technologies (29) because of its advantages over other techniques. These advantages, related to methodology and method application, are that the methodology is verifiable, understandable, and holistic (30); bridges the gap between qualitative and quantitative methods (31); has a controlled feedback process; and accommodates various statistical analysis techniques to interpret the data (32), among many others (33). However, it also has limitations. Li et al. (34) identified several limitations of the method, one of which is that a Delphi study is time-consuming to complete because the process includes multiple iterations or rounds. Additionally, the Delphi technique is very sensitive to design characteristics and the clarity of the question formulation. Furthermore, as the procedure depends on the quality of the feedback provided, the result must be carefully and responsibly analyzed (35).

To overcome the identified limitations, we modified the traditional Delphi process and propose the agile Delphi methodology, which will be explained in the “Research method” section.

## 3. Research method

This section explains the MAD, which adopts some original contributions designed to overcome the deficiencies identified in the traditional Delphi method. We modified the traditional Delphi method by adding specific techniques based on the agile philosophy to some steps of the traditional method. These modifications aimed to improve method efficiency and efficacy and overcome the limitations identified in the “Literature review” section.

**Figure 2:**
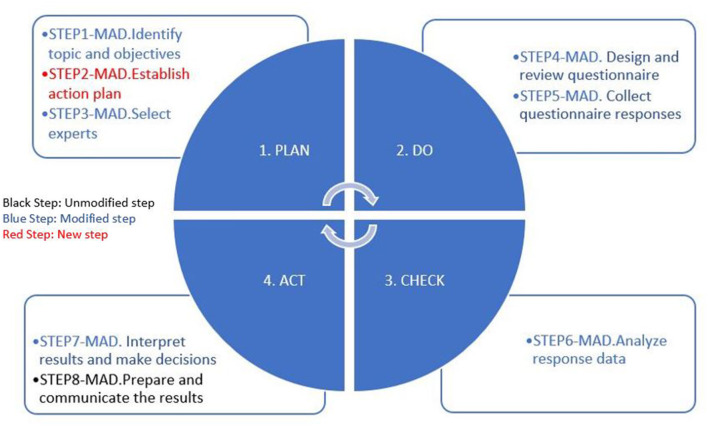
Agile Delphi methodology.

The transition from the traditional Delphi method toward an agile methodology requires a cultural change. We propose the use of the Plan-Do-Check-Act (PDCA) methodology that underpins the agile philosophy (36, 37). Figure 2 illustrates the agile Delphi methodology, where the agile methodology (PDCA) is combined with the steps of the traditional Delphi methodology. Each step of the agile methodology includes the unmodified steps of the traditional Delphi method (black type), the modified steps of the traditional Delphi method, including the techniques and mechanisms that we contributed (blue type), and new steps not existing in the traditional Delphi process but needed in MAD (red type).

This methodology, MAD, is agile (it follows an agile philosophy), iterative (it repeats the loop or cycle, called rounds in traditional Delphi, where three rounds are recommended), and incremental (it gradually increases the level of detail of the results about whether consensus is reached or not).

Agile Delphi methodology (MAD) defines two work teams:

- Delphi process owner team. This team is composed of three experts on OHS, one woman and two men who are experienced in OHS, specifically burnout syndrome. This team is involved in the following MAD methodology steps: identify the topic and objectives, establish the action plan, select and form the panel of experts, design the questionnaire, collect questionnaire feedback, review the modified questionnaire, interpret results, and make decisions.
- Delphi expert team. This team is composed of four Delphi process experts, two women and two men, who all have experience with and knowledge of methodologies and processes. This team is involved in the following MAD methodology steps: identify the topic and objectives; establish an action plan; design a questionnaire, review a questionnaire, collect feedback on the questionnaire, review the modified questionnaire, start communication, collect questionnaire responses, analyze response data, interpret results and make decisions, and prepare and communicate the results.

In addition, there are two well-established roles:

- Experts: A group whose responsibility is to issue judgments and opinions, which is the core of the method.
- Coordinator: A group that coordinates the process with a variable number of members ranging from two to five people. They coordinate each work team.

Each step of the agile Delphi methodology is described as follows: First, each step of the methodology is framed within the respective PDCA phase (36, 37) and then described according to the following descriptors:

- Type: either “traditional” if it was already part of the Delphi method, “modified” if an agile technique has been added to the Delphi method to carry out the step in MAD, and “new step” if this step was not previously part of the Delphi method.
- Description: description of the step.
- Activities: activities to be performed in the step.
- Teams and roles involved in the step.
- Deliverable: deliverable of the step.

### 3.1. Phase 1: Plan

The purpose of this phase is to describe the problem on which the experts are to reflect. For this purpose, the activities performed in this phase are to identify the topic and study objectives, establish the action plan, and select and form the panel of experts. The following lists the steps of the Plan phase.

#### 3.1.1. Step 1-MAD: Identify the topic and objectives

Type: Modified step.

Description: In this step, the topic, goal, and objectives are defined. The recommended technique for use in this step is SMART (ER) (38). The Delphi process owner team identifies the topic, the goal, and the objectives to be studied, making sure that the objectives are specific, measurable, achievable, relevant, time-bound, engaging, and rewarding.

Activities to be carried out:

- Activity 1: Briefly describe the topic.
- Activity 2: Identify the general goal.
- Activity 3: Break down the general goal into specific objectives.
- Activity 4: Check if each goal is SMART (ER).

Team and roles involved:

- Team: Delphi process owner team and Delphi expert team
- Role: Coordinator

Deliverable: Table 1 stores the information from this step.

**Table 1:** Description of the topic and objectives.

**Topic**							
Description of the topic:							
General objective:							
Specific objectives							
	S	M	A	R	T	E	R

#### 3.1.2. Step 2-MAD: Establish an action plan

Type: New step.

Description: In this step, the action plan is described, defining the activities to be developed as part of MAD, the timeline (start, finish), the person/s responsible for each activity, and deliverables. The recommended technique for use in this step is the GANTT chart (39).

Activities to be carried out:

- Activity 1: Define activities, sequence activities, estimate resources, estimate durations, and develop a schedule.
- Activity 2: Prepare the GANTT chart with the above information.
- Activity 3: Review the GANTT chart (both teams).
- Activity 4: Complete Table 2.

**Table 2:** List of experts.

**Name surname**	**Institution**	**Position**	**Qualifications**	**Experience**	**Email**
					
…					

Team and roles involved:

- Team: Delphi process owner team and Delphi expert team
- Role: Coordinator

Deliverable: A GANTT chart stores the information from this step.

#### 3.1.3. Step 3-MAD: Select and form the expert panel

Type: Modified step.

Description: The panel of experts who are to share their knowledge and experience for the study is selected and formed.

The Delphi process owner team identifies the potential experts that are to participate in the Delphi process. The topic to be studied must be taken into account to identify and select the most suitable experts. The expert selection criteria document is available at: https://www.promiseinnovatech.com/images/files/MAD-Methodology/ExpertNetwork.pdf.

Activities to be carried out:

- Activity 1: Define the areas of expertise.
- Activity 2: Define the typology of experts: type of expertise (occupational hazards, etc.), academic qualifications, and years of experience.
- Activity 3: Identify and brief experts. Select 15 to 20 experts on the subject under study from different disciplines. For topic evaluation, representativeness is based on the quality rather than the quantity of the experts.
- Activity 4: Contact the experts to ask them to participate in the MAD. To be able to confirm or refuse participation, they must be sent the following information: topic, description, and objectives of the process to be carried out, as well as the number of rounds to be implemented and the estimated time of the process.
- Activity 5: Complete Table 2.

Team and actors involved:

- Team: Delphi process owner team
- Actor: Coordinator

Deliverable: Table 2 stores the information from this step.

### 3.2. Phase 2: Do

The purpose of this phase is to conduct a questionnaire round. Therefore, the questionnaire for the round is designed, and the expert responses to the questionnaire are reviewed. The steps of the Do phase are detailed as follows.

#### 3.2.1. Step 4-MAD: Design and review the questionnaire

This step is composed of five sub-steps:

- Step 4.1 Design questionnaire.
- Step 4.2. Review questionnaire.
- Step 4.3. Collect feedback on the questionnaire.
- Step 4.4. Review the modified questionnaire.
- Step 4.5. Start communication.

##### 3.2.1.1. Step 4.1-MAD: Design questionnaire

Description: The Delphi process owner team designs the questionnaire for each of the rounds. The questionnaire must be aligned with the objectives specified in Step 1 and will include three types of questions:

1. Questions requiring numerical data as a response. For example, “How many hours do you spend answering email?”
2. Questions requiring categorical data as a response. For instance, “Rank your job search priorities (on a scale from 1—least important to 5—most important).
  [ ] Physical office location
  [ ] Working hours
  [ ] Telecommuting
  [ ] Social benefits (tickets to theme parks and health insurance)
  [ ] Salary”
3. Open-ended questions, that is, respondents can write whatever they regard to be relevant and give their own judgments.

Recommended number of questions: 15

Activities to be carried out:

- Activity 1: Design questions aligned with the specific objectives of the Delphi process. The questionnaire must include (i) sociodemographic questions to provide an overall description of the group of people answering the questionnaire, and (ii) questions that cover the specific objectives defined in Step 1. The questions must be closed-ended, precise, unambiguous, and understandable, phrased in a language that is appropriate and understandable for experts. Each question must refer to a single objective or concept; that is, there should not be two questions in one. Each question should be aligned with a specific objective. Questions should be placed in a logical order, from easiest to hardest.
- Activity 2: Design response. Depending on the questionnaire type, descriptive or analytic, the responses will be numerical values, categories, yes/no, an ordinal scale, or text (open-ended responses).
- Activity 3: Test or pilot the designed questionnaire with a small number of people to analyze the adequacy of each question and evaluate the clarity of the approach, number of questions, and intelligibility of the content.
- Activity 4: Make the appropriate modifications based on the conclusions of the test or pilot survey.
- Activity 5: Fill in Table 3.

**Table 3:** Draft questionnaire.

**Question 1:**	
Possible response scales:	
Specific objective covered:	
Question 2:	
Possible response scales:	
Specific objective covered:	
….	

Team and roles involved:

- Team: Delphi process owner team and Delphi expert team
- Actor: Coordinator

Deliverable: Table 3 stores the information from this step.

##### 3.2.1.2. Step 4.2-MAD: Review questionnaire

Description: The Delphi expert team reviews the questionnaire designed by the Delphi process owner team.

Activities to be carried out:

- Activity 1: Analyze the adequacy of each questionnaire item, taking into account the objectives established in Step 1.
- Activity 2: Analyze the clarity and approach of questions and answers, the number of questions, and the instructions for experts.
- Activity 3: Analyze which statistics to apply with each question. We are aware that not just any statistic can be used with any type of response. If the statistic that can be used for each questionnaire item has not been previously analyzed, it may not be possible to statistically analyze the data at the end of the MAD process. For this reason, we related the type of response to the statistic to be used to ensure that the results are analyzable.
- Activity 4: Recommend actions to solve the problems identified in the questionnaire.
- Activity 5: Fill in all fields of Table 4 except the “*Has it been done?*” section.

**Table 4:** Review of the draft questionnaire.

**Question 1:**	
Possible response scales:	
Response types:	
Statistics to be applied	
- Descriptive	
- Test	
Specific objective with which response is aligned	
Clarity/approach/	
…	
Actions to be carried out by the Delphi process owner team (Y/N)	Has it been done? (Y/N)
Action 1	

Team and actors involved:

- Team: Delphi expert team
- Actor: Coordinator

Deliverable: Table 4 stores the information from this step.

##### 3.2.1.3. Step 4.3-MAD: Apply feedback on the questionnaire

Description: The Delphi process owner team will review the Delphi process actions recommended by the expert team and make the necessary modifications to the questionnaire.

Activities to be carried out:

- Activity 1: Review Table 4 for each question.
- Activity 2: Carry out the recommended actions on each of the questionnaire items, modifying the questions or answers in Table 4.
- Activity 3: After applying feedback, fill in the “*Has it been done?*” section of Table 4 with *Y* (yes) or *N* (no). If *N* is entered, specify why the recommended actions have not been carried out.

Team and roles involved:

- Team: Delphi process owner team
- Actor: Coordinator

Deliverable: Fully completed Table 4 stores the information.

##### 3.2.1.4. Step 4.4-MAD: Review the modified questionnaire

Description: The Delphi expert team reviews the modifications made to the questionnaire and approves the final questionnaire.

Activities to be carried out:

- Activity 1: Review the modified questionnaire.
- Activity 2: Check if all the actions have been carried out.
- Activity 3: Discuss the recommended actions that were not carried out and decide on the final questionnaire.
- Activity 4: Fill in Table 5.

**Table 5:** Final questionnaire.

**Question 1:**	
Possible response scales:	
Specific objective covered:	
Question 2:	
Possible response scales:	
Specific objective covered:	
….	

Team and roles involved:

- Team: Delphi process owner team and Delphi expert team
- Actor: Coordinator

Deliverable: Table 5 stores the information.

##### 3.2.1.5. Step 4.5-MAD: Start communication

The Delphi expert team informs the panel that round x is starting and provides access to the questionnaire with the instructions for completion.

Activities to be carried out:

- Activity 1: Communicate the following information (entered in Table 6) to the experts: start date, finish date, how to access the questionnaire, the date on which the panel will be contacted, and instructions about how to answer the questionnaire.

**Table 6:** Record of the round.

**Start date**	
Finish date	
Information on how to access the questionnaire	
Date experts were notified	
Instructions on questionnaire completion	

Team and roles involved:

- Team: Delphi process expert team
- Actor: Coordinator

Deliverable: Table 6 stores the information.

#### 3.2.2. Step 5-MAD: Answer the questionnaire

The experts answer the questionnaire before the finishing date of the round. The Delphi process expert team checks if all the experts have answered the questionnaire and sends a reminder of the date on which the round ends 3 days before the end of the round.

Activities to be carried out:

- Activity 1: Answer the questionnaire (experts).
- Activity 2: Check that the experts are responding to the questionnaire and send a reminder of the deadline for questionnaire completion. Record the date on which they communicated with the experts in Table 7.

**Table 7:** Record of communication with experts.

**Start date**	
Finish date	
Information on how to access the questionnaire	
Date experts were notified	
Reminder for experts of the closing date of the round	

Team and actors involved:

- Team: Delphi experts team
- Actor: Coordinator and experts

Deliverable: Table 7 is completed by the coordinator of the agile Delphi process expert team.

### 3.3. Phase 3: Check

The purpose of this phase is to check the expert responses. To do this, the data are analyzed. The steps of the Check phase are explained as follows.

#### 3.3.1. Step 6-MAD: Analyze response data

The Delphi expert team conducts a statistical analysis of the expert responses to each of the questionnaire items.

Activities to be completed:

- Activity 1: Analyze the responses to each question. Some of the possible statistics to be analyzed are as follows:
  - Mean, median, mode, maximum, minimum, standard deviation, and quartiles
  - The interquartile range: RQ = Q3 – Q1
  - Relative interquartile range RIR = (Q3 – Q1)/Q2 (median)
  - Percentage of responses in the range of the median ± 1
  - Kendall rank correlation coefficient
  - Chronbach's alpha

- Activity 2: Provide summary statistics and plot graphs.

Team and roles involved:

- Team: Delphi expert team
- Actor: Coordinator

### 3.4. Phase 4: ACT

The purpose of this phase is to interpret the results. In addition, the Delphi expert team decides, based on the results, whether another round is needed, and the results of this round are reported to the experts. The steps of the Act phase are detailed as follows.

#### 3.4.1. Step 7-MAD: Interpret results and make decisions

The Delphi expert and process owner teams analyze the results. They make decisions, interpret responses, and evaluate the actions to be taken in the next round.

Activities to be carried out:

- Activity 1: Interpret statistical results.
- Activity 2: Evaluate decision-making.
- Activity 3: Complete Table 8, detailing, for each objective identified in Step 1, the conclusions of the data analysis and results from interpretation, and the decision-making on the next round.

**Table 8:** Conclusions and decision making.

**Specific objective 1**	
Conclusions	
Decision making	
Specific objective 2	
Conclusions	
Decision making	
…	

Team and actors involved:

- Team: Delphi process owner team and Delphi expert team
- Actor: Coordinator

#### 3.4.2. Step 8-MAD: Prepare and report results

The Delphi process expert team reports the results and notifies the experts ahead of the next round.

The activities to be carried out:

- Activity 1: Give feedback to the experts and design the questionnaire for the next round.
- Activity 2: Communicate the feedback obtained from the round to the experts. Feedback should include information on the responses from the previous round, considering the type of questions asked in the respective round.

Team and actors involved:

- Team: Delphi expert team
- Actor: Coordinator

At the end of a PDCA cycle, an assessment of whether or not it is necessary to carry out a new round is conducted. If so, another PDCA cycle starts, with the exception that Phase 1 is not carried out from scratch: the objectives, experts, and action plan are reviewed, taking into account the actions of the previous round.

To apply the MAD methodology, companies do not need to invest in a specific tool. The methodology is designed for use with a spreadsheet and a form for the questionnaires. These are both tools that are fully accessible to companies as part of a Google or Microsoft package.

## 4. Case study

Now that the MAD methodology has been defined, we have to measure its efficiency against the traditional Delphi method. For this purpose, we developed a case study to test MAD. This section reports several findings related to:

- The comparison of the agile Delphi methodology (MAD) with the traditional Delphi methodology.
- The results of applying MAD to analyze whether SMEs are considering burnout syndrome as a psychosocial risk, what factors affect or increase employee burnout, and what mechanisms could be implemented to mitigate and reduce burnout syndrome at the workplace.

### 4.1. Research design

In this case study, three MAD rounds were carried out:

Round 1: The questionnaire was composed of nine questions. There were seven questions based on the sociodemographic and professional characteristics of the participants and two multiple-choice questions to check what the OHS risks and benefits of digital transformation are and discover if burnout is one. The estimated questionnaire response time was 6 min. Experts had 8 days to respond online and received one reminder by email.

Round 2: The questionnaire was composed of eight questions. The questions were devised to discover whether there was consensus about the following:

- Whether enterprises consider burnout syndrome to be a psychosocial risk?
- What ICT and non-ICT mechanisms mitigate burnout syndrome?
- What factors affect this syndrome?

The estimated questionnaire response time was 8 min.

Round 3: The questionnaire was composed of nine questions. The estimated questionnaire response time was 15 min. The questions asked in this round were designed to prompt deeper reflections from experts on the factors and mechanisms that affect and mitigate burnout syndrome.

Participants: A total of 16 experts, experienced and knowledgeable in occupational risk prevention, participated; 56% were women and 44% were men. More than 90% of the experts were in the over 45 year age group, which denotes the extent of expert knowledge and experience. Participation was voluntary and kept confidential throughout the study. The participants do not know each other or know who is participating in the process.

Research questions: The research questions are shown in Table 9.

**Table 9:** Research questions.

**Topic**	**Topic**	**Research questions**
A. Agile Delphi	Compare traditional Delphi vs. agile Delphi	Question 1.A: Is the agile Delphi methodology really agile? Is MAD faster than traditional methodology? How long did it take to make a case with both methodologies? Question 2.A: What was the rework effort required to align the questionnaire items with the identified objectives?
B. Burnout syndrome	Psychosocial risk	Question 1.B: Do enterprises consider burnout syndrome to be a psychosocial risk? As OHS experts, do you consider burnout to be a psychosocial risk?
	Technology affects burnout syndrome	Question 2.B: Do companies have data to corroborate that ICTs have a negative impact on burnout syndrome, that is, technological resources and tools have a negative effect and help to increase burnout syndrome among employees? Question 3.B: Do companies have ICT mechanisms in place that help to reduce burnout syndrome?
	Factors that increase burnout syndrome	Question 4.B: Which factors do experts currently consider to have the greatest impact on burnout syndrome at companies?
	Mechanisms to mitigate burnout syndrome	Question 5.B Does the company you work for have mechanisms to prevent and reduce burnout syndrome? Question 6.B. Which ICT mechanisms are more effective for companies to prevent burnout syndrome? Question 7.B Which non-ICT mechanisms are more effective for companies to prevent burnout syndrome?

ICT, information and communication technology.

Abbreviation: ICT for information and communication technology.

Results: The results are reported in Sections A and B below.

#### 4.1.1. (A) Results of applying the agile Delphi methodology vs. the traditional Delphi method

We carried out two independent studies: Case Study A (CS-A) following the traditional Delphi method and Case Study B (CS-B) following the agile Delphi methodology:

- The traditional Delphi method followed the iterative method that repeats the steps to try to reach a consensus among the experts on a topic, in this case, burnout syndrome.
- The agile Delphi methodology took an agile approach, following the agile, iterative, and incremental methods explained in the “Research method” section.

Although the participants in the studies were different people, both studies were conducted according to the same research design using the same number of participants with the same profiles.

In CS-A, the Delphi expert team was composed of researchers from the Universidad Carlos III de Madrid belonging to the IRSST-UC3M Chair: R&D for Intelligent Digital Transformation of Occupational Health and Safety. The Delphi process owner team was composed of two researchers from Universidad Carlos III de Madrid who did not belong to the IRRST-UC3M Chair.

In CS-B, the Delphi expert team was composed of researchers from Universidad Carlos III de Madrid belonging to the IRSST-UC3M Chair: R&D for Intelligent Digital Transformation of Occupational Health and Safety. The Delphi process owner team was composed of two experts in burnout syndrome from the Madrid Regional Health and Safety at Work Institute (IRSST) in Spain.

The number of experts in both cases was 14 for the CS-A group and 16 for the CS-B group. In both cases, the participant profile included experts in occupational risk prevention. The research design was the same (number of rounds and expert profile). The tools used to analyze the data and run the survey were Microsoft Excel and Microsoft Forms, respectively.

Several questions were formulated in order to compare both methods and check if the results of the agile Delphi methodology were better than those of the traditional Delphi method.

Question 1.A: Is the agile Delphi methodology really agile? Is MAD faster than the traditional methodology? How long does it take to build a case with the traditional Delphi method compared with the agile Delphi methodology? To check the time taken, we analyzed the planning of both case studies. Table 10 shows a comparison of both case studies. The panel of experts was different for each case study to avoid bias, but both had similar experience and profiles. Both case studies began on the same date, that is, 1 March (they were developed simultaneously), but CS-B (using MAD) ended almost a month earlier. Therefore, we can state that it takes less time to run the agile Delphi methodology than the traditional methodology. The activities that took less time were the steps for which we had recommended techniques or created mechanisms to improve their efficiency.

**Table 10:** CS-B: agile Delphi methodology.

**Case study A—traditional Delphi method**					**Case study B—agile Delhi methodology**					
**Round**	**Step**	**Start date**	**Finish date**	**Days**	**Round**	**Phase**	**Step**	**Start date**	**Finish date**	**Days**
1	STEP1-T	03/01/2022	03/18/2022	14	1	1. Plan	STEP1-MAD	03/01/2022	03/11/2022	9
	STEP2-T	03/21/2022	04/20/2022	23			STEP2-MAD	03/14/2022	03/18/2022	5
	STEP3-T	04/21/2022	04/29/2022	7			STEP3-MAD	03/21/2022	04/20/2022	23
	STEP4-T	04/29/2022	05/05/2022	4		2. Do	STEP4-MAD	04/18/2022	04/25/2022	6
	STEP5-T	05/05/2022	05/06/2022	7			STEP5-MAD	04/26/2022	04/29/2022	4
	STEP6-T	05/06/2022	05/09/2022	2		3. Check	STEP6-MAD	05/04/2022	05/04/2022	2
	STEP7-T	05/09/2022	05/09/2022	1		4. Act	STEP7-MAD	05/04/2022	05/04/2022	1
	–	–	–	–			STEP8-MAD	05/05/2022	05/05/2022	1
2	STEP1-T	05/09/2022	05/10/2022	2	2	1. Plan	STEP1-MAD	05/05/2022	05/05/2022	1
	STEP2-T	05/10/2022	05/10/2022	1			STEP2-MAD	05/05/2022	05/05/2022	1
	STEP3-T	05/10/2022	05/17/2022	6			STEP3-MAD	05/05/2022	05/05/2022	1
	STEP4-T	05/17/2022	05/20/2022	4		2. Do	STEP4-MAD	05/06/2022	05/09/2022	4
	STEP5-T	05/23/2022	05/27/2022	5			STEP5-MAD	05/10/2022	05/16/2022	5
	STEP6-T	05/29/2022	05/31/2022	2		3. Check	STEP6-MAD	05/16/2022	05/17/2022	2
	STEP7-T	06/01/2022	06/01/2022	1		4. Act	STEP7-MAD	05/17/2022	05/18/2022	2
	–	–	–	–			STEP8-MAD	05/19/2022	05/19/2022	1
3	STEP1-T	06/01/2022	06/01/2022	1	3	1. Plan	STEP1-MAD	05/20/2022	05/20/2022	1
	STEP2-T	06/01/2022	06/01/2022	1			STEP2-MAD	05/20/2022	05/20/2022	1
	STEP3-T	06/02/2022	06/08/2022	5			STEP3-MAD	05/20/2022	05/21/2022	2
	STEP4-T	06/09/2022	06/14/2022	4		2. Do	STEP4-MAD	05/21/2022	05/22/2022	2
	STEP5-T	06/15/2022	06/22/2022	6			STEP5-MAD	05/23/2022	05/27/2022	5
	STEP6-T	06/22/2022	06/24/2022	3		3. Check	STEP6-MAD	05/30/2022	05/31/2022	2
	STEP7-T	06/27/2022	06/27/2022	1		4. Act	STEP7-MAD	06/01/2022	06/02/2022	2
	–	–	–	–			STEP8-MAD	06/03/2022	06/03/2022	1

**Figure 3:**
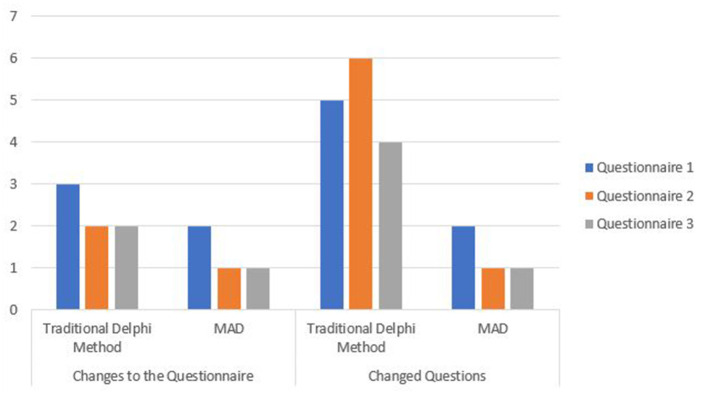
Rework.

Question 2.A: What is the quality of the questionnaire? How many times do you have to redo the questionnaire or rework questions? Not only is it important to be more efficient from the point of view of the time spent performing the Delphi process, but it is also necessary to check the quality of the questionnaires. The question that we formulated was, “What rework effort was required in order to align the questionnaire items with the identified objectives?” To answer this question, we checked how much rework was required on the questionnaire for each round. Rework means how many times, on average, the questionnaire items had to be redone or revised before they were considered to be complete. As Figure 3 shows, the number of reworks was lower using the techniques and mechanisms defined in MAD, leading to higher-quality questionnaires in fewer iterations.

#### 4.1.2. (B) Results of the burnout syndrome study using agile Delphi

The dossier containing the information gathered at each step of the MAD methodology during case study development is available at https://www.promiseinnovatech.com/products/other-research-outputs.

Psychosocial risk: Although burnout syndrome occupies a prominent place on the list of psychosocial occupational risks, this research was conducted to find out if there is consensus among OHS experts on the following questions.

Question 1.B: Do enterprises consider burnout syndrome to be a psychosocial risk? As an OHS expert, do you consider burnout to be a psychosocial risk? The results in terms of consensus among OHS experts are clear and compelling: 75% of the enterprises employing the experts do consider burnout syndrome to be a psychosocial risk. In addition, there is a unanimous agreement among the experts that burnout syndrome is a psychosocial risk and that OHS must consider it as such.

Technology affects burnout syndrome: In view of the exponential growth of both digital technologies and burnout syndrome, we raised the following questions.

Question 2.B: Do companies have data to corroborate the fact that ICTs have a negative impact on burnout syndrome, that is, that technological resources and tools have a negative effect and help to increase burnout syndrome among employees? A total of 25% of the companies have data that support that ICTs negatively affect worker burnout syndrome.

Question 3.B: Do companies have mechanisms to mitigate the negative impact of ICTs on burnout syndrome, that is, technological resources and tools to help reduce burnout syndrome among employees? A total of 13% of companies do have mechanisms. Although this is a rather low percentage, it is encouraging in the sense that there is hope that ICTs can be used as a lever to reduce burnout syndrome symptoms.

Factors that increase burnout syndrome: There are a lot of studies exploring the factors that affect burnout syndrome. However, these factors have not been studied from an occupational risk prevention perspective. In this study, we formulated the following question.

Question 4.B: Which factors do experts currently consider to have the greatest impact on the increase in burnout syndrome among employees at the workplace?

**Figure 4:**
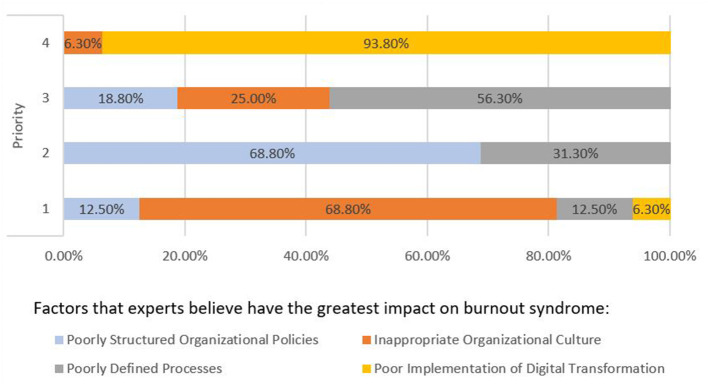
Consensus on factors.

Figure 4 shows the degree of consensus for the effect of each factor on burnout syndrome (Option 1—the most and Option 4—least) as prioritized by the experts. The y-axis shows the priority. For example, the percentage of experts that consider burnout syndrome to be exacerbated by an inappropriate organizational culture is 68.80%, 12.50% for poorly structured organizational policies, 12.50% for poorly defined processes, and only 6.30% for poor implementation of digital transformation. This result was elicited in round 2. Once the data had been analyzed, this information was delivered to experts in the next round (round 3). The objective of this feedback was for experts to reflect on their responses, considering the opinions of the other experts. In round 3, they were asked the same question to check if they had changed their opinion. They were also asked to think more specifically about each of the factors and provide specific actions to mitigate their effect on burnout. The actions identified for each factor are shown in Table 11.

**Table 11:** Actions for each factor.

**Factor: poorly structured organizational policies**
- Have innovative, flexible, mobile, open, clear policies - Define lines of authority - Enable communication and autonomy between departments - Identify each position, its function and to whom it reports
**Factor: inappropriate organizational culture**
- Have well-defined company vision, mission, and values - Set goals - Have a common purpose and direction - Provide a strategy alignment: coherence between directives and managerial actions
**Factor: poorly defined organizational processes**
- Define activities - Distribute workload appropriately - Assure competent leadership - Change management skills
**Factor: poorly implemented digital transformation**
- Provide change management skills - Set store by the person and not just technology - Avoid depersonalization and lack of human contact - Reduce complexity and constant changes of technologies - Distribute workload appropriately - Assure competent leadership - Provide continuous training and development of technological skills - Allow for adaptation and learning time - Provide troubleshooting support - Use non-intrusive tools to prevent employees feeling that they are being constantly monitored and observed

Mechanism to mitigate burnout syndrome: Having identified the business factors that affect employee burnout syndrome, the next step was to ask the experts different questions related to reducing the impact of the identified factors. Do the companies have mechanisms in place to prevent and mitigate burnout? Taking into account that enterprises need both ICT and non-ICT mechanisms to mitigate burnout syndrome, which mechanisms, based on your knowledge and experience as an OHS specialist, are more effective for companies to prevent burnout syndrome?

Question 5.B: Does the company you work for have mechanisms to prevent and reduce burnout syndrome? A total of 63% of experts agreed that their enterprise did not have mechanisms in place, 13% stated that their enterprises did, and 25% said N/A (do not know or not applicable).

Question 6.B: Which are the most effective ICT mechanisms to prevent burnout syndrome at companies?

- ICT mechanisms that favor adequate and effective communication channels for relationships between workers, middle managers, and managers of the organization.
- Technological resources are adapted to jobs to facilitate the routine work of its employees. If this is not the case, technological media cease to be facilitators and become inhibitors, which is one of the causes that provoke burnout syndrome.
- Continuous training. ICTs are constantly evolving, and employees need to be prepared for change. The only way to do this is through continuous training and online help systems.
- ICT mechanisms to detect symptoms of mental fatigue and evaluate the state of the worker.
- Digital disconnection tools and protocols. These frameworks will guarantee the right of workers to effectively enjoy their rest time as well as the right to preserve their personal and family privacy.

Question 6.B: Which are the most effective non-ICT mechanisms to prevent burnout syndrome at companies?

- Work autonomy indicates that employees have time flexibility and can make decisions about how to organize their work.
- Implementation of psychosocial and physical health strategies, like mentoring techniques and training to develop skills for emotional management on top of change management techniques from a psychosocial perspective.
- Teamwork promotion, through the improvement of communication and the creation of safe spaces where communication is encouraged.
- Worker reporting and action channels and protocols.

## 5. Conclusion

Although burnout is not a new syndrome, having been around for more than 40 years now, it is true that the number of cases has increased alarmingly in recent years, to the point that it is now considered to be the “second” or “silent” pandemic. Many studies have been conducted from the point of view of burnout sufferers. However, as psychiatrist Christina Maslach pointed out, we should not overlook the fact that, in the case of burnout syndrome, the organization rather than the individual is at fault; that is, burnout is the responsibility of the employer. To help companies reduce this syndrome, we carried out a study using the Delphi method, in which experts who have extensive knowledge and experience of burnout syndrome participated. The goal of the study was to reach a consensus on the factors that cause burnout and the mechanisms that should be used at companies to mitigate the syndrome among their employees. While there are other works with the same goal, this research is original in that we have used a method where the participants, recognized, knowledgeable, and experienced experts in burnout syndrome, have, across several iterations or rounds, reached a consensus on their responses. They have had the opportunity to reflect on both their own opinions and the viewpoints of the other experts and, if appropriate, modify their own responses in the next round. The traditional Delphi method has several limitations. For instance, it is time-consuming and has shortcomings with respect to the definition of questionnaires and statistics. To overcome these shortcomings, we defined the agile Delphi methodology (MAD), which combines an agile methodology with the traditional Delphi method. To do this, we added some steps and modified others. Additionally, we defined mechanisms or recommended techniques to make the method steps more agile and efficient.

The limitation of this study is that all the participating experts were Spanish. Although these experts are well-acquainted with burnout syndrome in the Spanish context, we cannot be certain that, being a psychosocial risk, it will affect workers equally regardless of the country in which they perform their work activity. To be sure that the results are independent of the country in which the study is carried out, it would have to be replicated in different countries or with experts of different nationalities. In this case, the results would be more generalizable globally. The findings of this study were validated in two ways: one validation compared the agile Delphi methodology (MAD) and the traditional Delphi methodology to check if MAD is more agile and efficient. As the results reported in the “Case study” section show, we can state that the agile Delphi methodology (MAD) is more agile and efficient than the traditional Delphi methodology. The time taken is reduced by increasing the quality of the products, that is, the questionnaires, in fewer iterations. For the other validation, OHS experts identified and discussed the factors causing an increase in burnout syndrome and the mechanisms to mitigate the effects of the identified factors. In this case, experts reached a consensus on the factors that most affect burnout, which are inappropriate organizational culture, poorly structured organizational policies, poorly defined organizational processes, and poorly implemented digital transformation. To help mitigate these factors, experts identified ICT mechanisms, such as creating communication channels, adapting technology to jobs, providing continuous training, online help systems, and digital disconnection tools, and non-ICT mechanisms, such as adopting work autonomy, as well as flexibility and conciliation protocols, and developing psychosocial and physical health strategies.

The next step in this research would be to implement the identified mechanisms at organizations and evaluate whether, as the experts claim, the burnout syndrome rate decreases.

## Data availability statement

The raw data supporting the conclusions of this article will be made available by the authors, without undue reservation.

## Ethics statement

Ethical review and approval was not required for the study on human participants in accordance with the local legislation and institutional requirements. Written informed consent from the participants was not required to participate in this study in accordance with the national legislation and the institutional requirements.

## Author contributions

FM-D has designed the work presented in this paper and guided its development. FM-D, M-IS-S, G-LD-P, AA-S, and SV have participated in the reviewing process, analysis, synthesis of the case study summarized in this paper, and writing process. All authors contributed to the article and approved the submitted version.
